# Sources of Differences in On-Orbital Total Solar Irradiance Measurements and Description of a Proposed Laboratory Intercomparison

**DOI:** 10.6028/jres.113.014

**Published:** 2008-08-01

**Authors:** J. J Butler, B. C Johnson, J. P Rice, E. L Shirley, R. A Barnes

**Affiliations:** National Aeronautics and Space Administration, Goddard Space Flight Center, Greenbelt, MD; National Institute of Standards and Technology, Gaithersburg, MD 20899-0000; SAIC, Beltsville, MD

**Keywords:** absolute radiometric calibration, diffraction calculations, total solar irradiance (TSI), TSI uncertainty, TSI workshop, on-orbital TSI differences

## Abstract

There is a 5 W/m^2^ (about 0.35 %) difference between current on-orbit Total Solar Irradiance (TSI) measurements. On 18–20 July 2005, a workshop was held at the National Institute of Standards and Technology (NIST) in Gaithersburg, Maryland that focused on understanding possible reasons for this difference, through an examination of the instrument designs, calibration approaches, and appropriate measurement equations. The instruments studied in that workshop included the Active Cavity Radiometer Irradiance Monitor III (ACRIM III) on the Active Cavity Radiometer Irradiance Monitor SATellite (ACRIMSAT), the Total Irradiance Monitor (TIM) on the Solar Radiation and Climate Experiment (SORCE), the Variability of solar IRradiance and Gravity Oscillations (VIRGO) on the Solar and Heliospheric Observatory (SOHO), and the Earth Radiation Budget Experiment (ERBE) on the Earth Radiation Budget Satellite (ERBS). Presentations for each instrument included descriptions of its design, its measurement equation and uncertainty budget, and the methods used to assess on-orbit degradation. The workshop also included a session on satellite- and ground-based instrument comparisons and a session on laboratory-based comparisons and the application of new laboratory comparison techniques. The workshop has led to investigations of the effects of diffraction and of aperture area measurements on the differences between instruments. In addition, a laboratory-based instrument comparison is proposed that uses optical power measurements (with lasers that underfill the apertures of the TSI instruments), irradiance measurements (with lasers that overfill the apertures of the TSI instrument), and a cryogenic electrical substitution radiometer as a standard for comparing the instruments. A summary of the workshop and an overview of the proposed research efforts are presented here.

## 1. Introduction

**Fig. 1:**
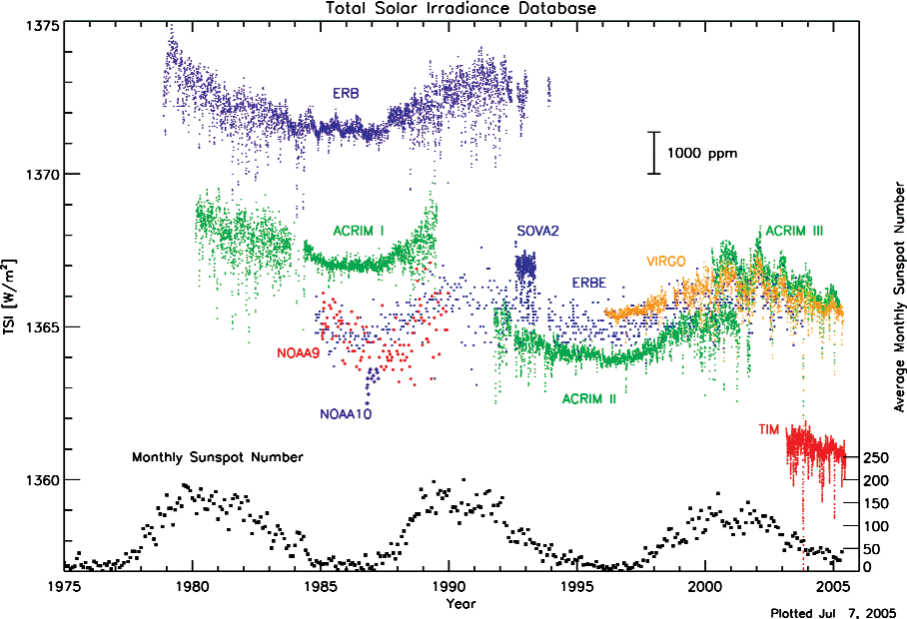
The 2005 TIM value for absolute TSI was about 1361 W/m^2^, whereas the ACRIM III and VIRGO (DIARAD + PMO6V) absolute TSI values are about 1366 W/m^2^ during the same time. The proposed work aims to understand this difference. (Graphic adapted from Greg Kopp’s presentation entitled “TIM Accuracy,” presented at TSI Uncertainty Workshop atr NIST, July 2005.)

The range of absolute total solar irradiance (TSI) values measured by different exo-atmospheric radiometers is currently about 5 W/m^2^, which is about 0.35 % (3500 × 10^−6^, Fig. 1) of the exo-atmospheric absolute TSI value at a distance of 1 astronomical unit (AU) from the Sun. This difference is greater than the individual standard uncertainties reported for most of these instruments, and greater than the 0.02 % per decade value typically stated as required to understand solar vs. anthropogenic forcing in climate change. The discrepancy between different instruments during the same time indicates the presence of unknown systematic bias. This motivated a National Aeronautics and Space Administration (NASA)-sponsored workshop on TSI uncertainty at the National Institute of Standards and Technology (NIST) in July 2005 that was attended by all current TSI instrument teams. Principle investigators were present from the Earth Radiation Budget Experiment (ERBE), the Active Cavity Radiometer Irradiance Monitor (ACRIM) I, II, III series, the Variability of solar IRradiance and Gravity Oscillations (VIRGO) DIfferential Absolute RADiometer (DIARAD) and the VIRGO Physikalisch-Meteorologisches Observatorium (PMO) 6V (PMO6V), and the Solar Radiation and Climate Experiment (SORCE) Total Irradiance Monitor (TIM) instrument.

The stated goals of the TSI Workshop were to 1) Identify and assess potential sources of current differences in on-orbit TSI measurements, and 2) Recommend measurement and algorithm-based approaches to address those differences. The 2.5 day agenda included detailed examination of the pre-flight and on-orbit measurement uncertainties of the instruments, careful consideration of the uncertainties and capabilities of on-orbit and ground-based instruments, discussions of an aperture-area intercomparison in progress, results of comprehensive diffraction analysis by NIST, and assessment of possible laboratory comparison measurements based on current measurement capabilities.

One hypothesis for the difference that was identified at the workshop involves the way in which scattering is controlled. This depends on the order of the defining aperture and the field-of-view limiting aperture. Another hypothesis for the difference is that it results from the novel frequency-domain-based power analysis approach used by TIM, as opposed to the traditional time-domain-based approach used by all other instruments. These hypotheses will be elaborated below. A laboratory intercomparison was proposed during the workshop, and is described here, which would enable both of these hypotheses to be tested and would check the system-level TSI scale of each participating instrument against NIST radiometric measurement scales.

## 2. Instruments

### 2.1 Common Features

All of these instruments measure total solar irradiance outside of the Earth’s atmosphere using the same fundamental method, that of the active cavity radiometer. Such instruments work on the principle of electrical substitution and have been reviewed extensively [1–3]. A circular aperture, typically 5 mm to 8 mm in diameter, is used to define a beam of sunlight that is subsequently absorbed in a black, metallic, thermally-isolated cavity. The temperature difference between this absorbing cavity and a non-illuminated cavity is actively controlled, and the additional electrical heater power required to maintain this temperature difference upon shuttering of the sunlight is measured. Except for several relatively small (generally <1 %) corrections, the ratio of this power measurement to the aperture area gives the value for total solar irradiance at the defining aperture. Spacecraft ephemeris information is then used to correct this to a Sun-to-instrument distance of 1 AU.

### 2.2 Design Differences

The design differences between the TSI active cavity radiometers tend to be in the details such as the number of cavities, shapes of cavities, cavity coating materials, dimensions, electronics, sampling frequency, data-reduction algorithms, calibration strategies, and type of orbit [VIRGO orbits L-1, others are in low-Earth-orbit (LEO)]. There are several design differences discussed at the workshop that could at first thought be considered as potential sources of the 0.35 % TSI scale difference. In this section we mention such design differences, dismissing some but leaving others to be more fully elaborated in Sec. 3 below. There are probably many other small design differences which were ignored at the workshop because they are not considered to be likely explanations for the scale difference.

Cavity coatings and designs differ amongst the TSI instruments. Instruments such as the ACRIM series and ERBE use cavities coated with a specular black paint and a geometry designed to trap the specular reflections. For VIRGO, PMO6V is constructed using an inverted cone with specular black paint, and DIARAD cylinder interiors are painted with a diffuse black paint. TIM utilizes, for the first time with space-based TSI measurements, nickel-phosphorous (NiP), a metallic black coating believed to be more robust against ultraviolet (UV) irradiation than black paints. NiP is a diffuse coating with very low (< 1 % across the solar spectrum) total reflectance. The effect of cavity shape for a diffuse coating, or for the residual diffuse reflectance of a specular coating, is to reduce the solid angle for scattering out of the first bounce. This is usually the ultimate limit of the cavity reflectance, regardless of whether the coating is specular or diffuse. The ACRIM team had the reflectance of some of their early cavities measured, and the TIM team measured the TIM cavity reflectance pre-flight extensively and monitored degradation using photodiodes on-orbit. Based on the careful design and measurements performed, with appropriate corrections apparently already made, the cavity coating and design differences probably are not responsible for the 0.35 % TSI scale difference.

There are other notable design differences that more likely can account for the scale differences. Compared with the other TSI instruments, TIM reverses the order of the two beam-limiting apertures that are in front of the cavity. This affects diffraction and scattering in subtle ways that seemed to not have been appreciated by all participants prior to the workshop. More will be said about this in Sec. 3.

Effects at the edges of apertures could also be a difference, though little was revealed in the workshop about the aperture edges from most of the existing instruments, except that the TIM apertures are evidently quite good knife edges. From one point of view, ideally an aperture would be beveled to a very sharp knife-edge. Any residual dullness, or a designed-in flat section, known as a “land’’, would create a surface within the aperture opening that would allow solar rays to reflect into the cavity rather than be clearly rejected. This could lead to difficult-to-analyze scattering issues when viewing solar radiation, and make the geometric area of the aperture difficult to measure with optical methods such as used by the NIST aperture measurement facility [4]. From another point of view, as pointed out by Claus Fröhlich from the VIRGO PMO6V team, a very sharp aperture is very thin near its edge, and thus could be susceptible to heating by the solar radiation, producing an extra infrared signal emitted by the aperture at the shutter frequency and absorbed by the cavity. Any of these effects would be captured by the laboratory intercomparison discussed in Sec. 4 below.

TIM also has a completely new algorithm by which it deduces the power measurement from the raw data. While all other instruments do this analysis in the time-domain, TIM data are analyzed in the frequency domain. As with all design differences, in principle it should not matter, but there are subtleties in practice that could lead to small effects. This also will be discussed further in Sec. 3.

Finally, there are differences relating to the treatment of on-orbit degradation. The general approach is to perform in-flight intercomparisons, either between different receiving cavities on the same instrument, or between different sensors in orbit at the same time. The source of the degradation can be related to solar exposure or independent of solar exposure, and some teams presented analytical models designed to represent these hypothesis, while other teams presented uncorrected and corrected ratios of intra-instrument cavity comparisons. Examples of solar exposure-related effects are changes in cavity reflectance and solar-induced thermal stresses. Other effects such as the shift from air to vacuum, exposure to the space environment, or simple lifetime performance may result in changes to the aperture area, thermal conductances, or electronic references (voltage, resistance, and gain).

Because the sensors are not retrievable and it is difficult to reproduce the operational environment in the laboratory, it is problematic to quantify and correct for on-orbit degradation. The estimates provided in the workshop varied between “absence of long term degradation’’ (ERBE) to 0.29 %/yr (PMO6V). However, as the TSI program has developed, the instruments have become more sophisticated with respect to quantifying on-orbit degradation. Incorporation of three or more identical cavities allows for a measurement protocol that results in one cavity receiving minimal exposure. TIM introduced the concept of independent monitoring of the cavity reflectance using silicon photodiodes and these data have proven useful. TIM also has the capability to perform on-orbit calibrations of the servo system gain and the stability of the thermal conductance is monitored using the time history of thermistor offsets. Intercomparisons among the four TIM cavities are used to track changes in the cavity reflectances, aperture areas, and the voltage and resistance standards.

### 2.3 Stated Uncertainties

One of the goals of the workshop was to come up with a complete list of the uncertainty budgets of the instruments. Without a statement of the uncertainty estimates for each TSI instrument, it would not be clear whether the observed 0.35 % difference in the mean TSI value between the instruments is outside of such uncertainties. At the workshop, the participants finalized a list of uncertainty components and supplied as many values as possible. The outcome of this effort is stated in Table 1. For the two instruments that do not correct for diffraction, the calculated magnitude (see Sec. 3.2) was taken to be the uncertainty. The on-orbit degradation value refers to the life of the mission. For most instruments the estimated *k* = 1 total uncertainty from such an analysis is below 0.1 % (1000 × 10^−6^). The uncertainty values in Table 1 are not necessarily correct, because these are estimates in many cases and some components that should be evaluated have not yet been included. The sense from most participants is that, even if these are somewhat underestimated, it is unlikely that the 0.35 % observed difference should be considered to be within the mutual uncertainty, as it seems statistically unlikely. Thus a search for systematic effects that could explain the scale difference is justified.

Because most of the TSI instruments have several different cavities, any of which are used to measure the Sun at a given time, it is possible to probe the difference in TSI values measured using different cavities within the same instrument, see Table 2. In Table 2, the “stated uncertainties’’ are values supplied by the participants for the different instruments. The “cavity variations’’ are one-half of the maximum difference between individual cavities on the same sensor. In some cases the cavity differences significantly exceed the expected uncertainty values (e.g., the assigned uncertainty to ACRIM III for diffraction should not be considered for intra-sensor cavity comparisons), indicating that indeed the stated uncertainty values reported in Table 1 are probably underestimated. In fact, it might be better to consider the spread in cavity differences to be a better indicator of uncertainty than the stated uncertainty budgets. Despite these issues, it is still believed that the 0.35 % scale difference is outside of reasonably conservative uncertainties, even those based on the cavity differences.

## 3. Potential Error Sources

### 3.1 Aperture Area

**Fig. 2:**
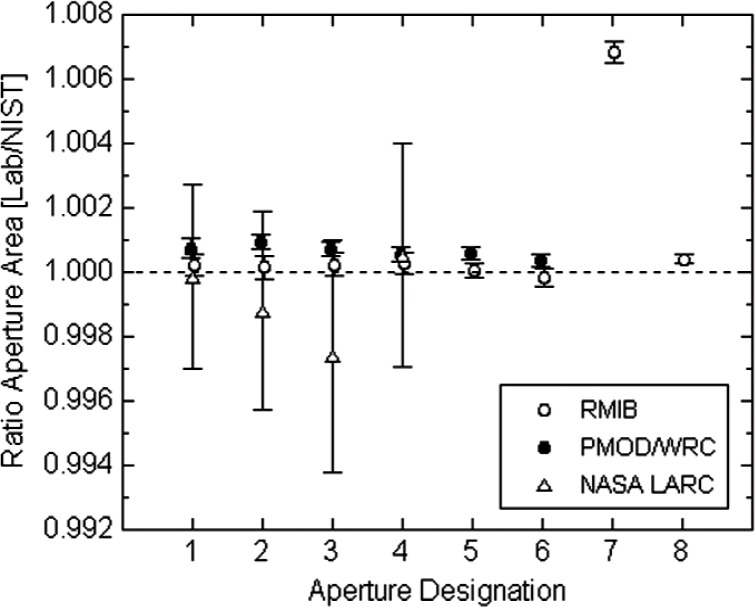
Results (to date, July 2005) from an aperture area intercomparison. Plotted is the ratio of each laboratory’s measurement of their own apertures to the NIST measurement of the same aperture. The uncertainty bars represent the expanded uncertainty *(k* = 2) of each measurement, and common “Aperture Designations” from different instruments as plotted on the ordinate actually represent different apertures. The Royal Metrological Institute of Belgium (RMIB) provided apertures representative of the VIRGO DIARAD instrument, and the World Radiation Center (WRC) provided apertures representative of the VIRGO PMO6V instrument. The NASA Langley Research Center (LARC) provided apertures representative of the Earth Radiation Budget Experiment (ERBE) instrument. For ACRIM, see the “note added in final preparation.” The TIM instrument had the areas of its flight apertures measured directly by NIST pre-flight, so its aperture area ratios to NIST would plot as a 1.0000 on this scale with a quoted uncertainty of 0.000025 (*k* = 1).

The defining aperture defines the diameter of the solar beam that enters the cavity. Its area must be accurately known. The geometric area of the defining aperture is the conceptually simplest error source that many would suspect of being responsible for the observed differences in TSI measurements. However, the initial results of a recent aperture area measurement intercomparison, as presented at the workshop by Carol Johnson of NIST and shown in Fig. 2, do not support such a hypothesis, based on the following reasoning. Though not obvious at first glance, the results in Fig. 2 can actually be viewed as the best available comparison between aperture areas of VIRGO and TIM. This is because the actual TIM flight apertures had been calibrated pre-flight directly against the NIST scale, using the same NIST aperture-area measurement facility that was subsequently used for the NIST intercomparison with VIRGO. As clearly seen in Fig. 2, representative (not flight) VIRGO aperture areas disagree with the NIST scale, and therefore the TIM scale, by no more than 0.1 %. Also, the direction of the disagreement is opposite to what would be needed to explain the 0.35 % differences in TSI measurements between VIRGO and TIM that were shown in Fig. 1. Therefore, based upon the evidence available to us at this time, it does not seem that the measured TSI differences arise from aperture area scale differences.

### 3.2. Diffraction

**Fig. 3:**
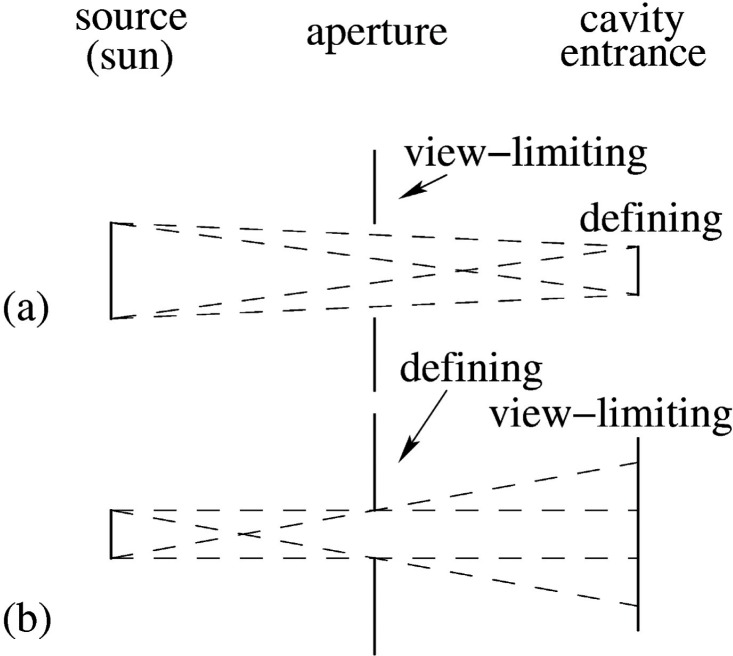
In most of the TSI instruments, ACRIM, ERBE, and the VIRGO instruments DIARAD, and PMO6V, the order of the apertures is as shown in (a) here, where there is a defining aperture immediately in front of the cavity and a view-limiting aperture towards the front of the instrument. The order of the apertures for the TIM instrument is as shown in (b) here, where instead the view-limiting aperture is the front of the cavity and the defining aperture is towards the front of the instrument.

Results of calculations of the effects of diffraction at the apertures of TSI instruments were recently made by Eric Shirley of NIST and presented by him at the workshop. These calculations were comprehensive in the sense that they applied the same formalism to all of the TSI instruments. For a review of the methods used with some examples of application to TSI instruments, see Ref. [5]. Table 3 provides a summary of the results. In order to effectively correct for diffraction, the geometric aperture area must be multiplied by the correction factor listed in Table 3. The direction of the diffraction correction for TIM is opposite (below unity) of that for the other instruments (above unity), because the order of the apertures for TIM is opposite of that for the other instruments. This geometry difference is illustrated in Fig. 3 and discussed further in the next section.

For the data shown in Fig. 1, the correction value listed has been applied to the TIM data, and correction values that had been computed independently and which agreed well with the values in Table 3 have been applied to the VIRGO data for instruments PMO06V and DIARAD. However, this correction has not been applied to the ERBE and ACRIM data shown in Fig. 1. Note that if it were applied to the ACRIM III data, for instance, it would lower the TSI data by about 0.13 %, making them lay almost halfway between the VIRGO and TIM data rather than in agreement with the VIRGO data as shown. Thus diffraction may account for one-third of the difference between TIM and ACRIM, but none of the difference between TIM and VIRGO.

### 3.3 Scattering

The order of the defining and the view-limiting apertures is opposite between TIM and the other TSI instruments, as illustrated in Fig. 3 and implied from the aperture dimensions listed in Table 3. The defining aperture is the one that defines the diameter of the solar beam that is supposed to enter the cavity, whereas the view-limiting aperture only limits the angular field of the view. In the arrangement used by most instruments, Fig. 3a, both the view-limiting and the defining apertures are illuminated by sunlight, but only a fraction of the rays entering the view-limiting aperture also enter the defining aperture and are measured. The rest of the rays must be captured by one or more baffles located between the view-limiting and defining apertures. However, some, albeit a small fraction, of these unwanted rays will scatter off of the baffles or the solar-illuminated view-limiting aperture and enter the defining aperture, where their power will be included with that of the directly illuminating beam. This effect, if not entirely corrected, would tend to make instruments that use the arrangement of Fig. 3a overestimate the solar irradiance compared to the instruments that used the arrangement depicted in Fig. 3b. This is because in Fig. 3b all of the solar rays pass through the defining aperture and the view-limiting aperture, so that the baffles between these two apertures are not illuminated by sunlight and are not susceptible to this scattering effect. Because TIM is the only instrument that uses the arrangement in Fig. 3b, whereas all other TSI instruments use the arrangement in Fig. 3a, this effect could explain the observed *direction* of the difference in measured TSI values.

It is not clear yet that this effect can account for the *magnitude* of the TSI difference. This is because some instrument teams claim to already have corrected for scattering in their instruments. It was not clear from the presentations at the workshop, however, that all teams had properly performed the correction for the specific type of scattering stated here. It was generally acknowledged, through the course of discussions at the workshop, that a uniform treatment of the scattering problem across all TSI instruments, analogous to what was done for diffraction, has not been performed but is recommended. It was also pointed out that this may be much more difficult to achieve in practice than it was for diffraction, because the scattering properties of the baffle and aperture materials are not necessarily known sufficiently. As a practical alternative, the laboratory inter-comparison proposed and described below in Sec. 4 will probe the scatter effect (along with the diffraction effect) experimentally, because it will measure instrument response both with beams that overfill and under-fill the view-limiting aperture of Fig. 3a.

### 3.4 Thermal Background

When the shutter in a TSI instrument is closed, there is still radiant power collected in the cavity that results from the thermally-emitted infrared radiance from the back of the shutter, apertures, baffles, etc., that gives rise to a small thermal background offset signal. The correction required for this effect is primarily from a thermal model for some instruments (ACRIM), possibly backed up by a one-time measurement, whereas it is from frequent, ongoing measurements for others (TIM). The TIM instrument measures the effect by repositioning the instrument to view deep space, where it views what is effectively a true zero with its shutter open, and then directly measures the thermal background offset with its shutter closed. The workshop did not go rigorously into the details of the way that each instrument determines the correction for this effect, so it was not clear whether or not it could be a source of the 0.35 % TSI scale difference.

### 3.5 Power Application

Most TSI instruments supply the electrical substitution heater power from a variable-level dc electrical source. However, the TIM instrument supplies it as a series of constant-level pulses, where the time-integrated level is varied using pulse-width modulation (PWM). During analysis of the first few months of SORCE TIM data, the TIM instrument team realized that a correction due to a specific type of nonlinearity was necessary due to the PWM scheme. They were able to characterize a ground TIM unit with a copy of the PWM circuit in order to determine the non-linearity correction, but because of possible unit-to-unit dissimilarity, had to increase the overall TIM uncertainty slightly when applying the correction to the flight unit. This added component of uncertainty was primarily responsible for de-rating the overall SORCE TIM standard uncertainty from the predicted 0.01 % value to 0.03 %. Unless the non-linearity correction procedure developed by the TIM team is somehow flawed, which does not seem likely due to the straightforward nature of the electrical measurements involved, this effect is not nearly large enough to account for the 0.35 % TSI scale difference.

### 3.6 Power Demodulation

The TIM phase-sensitive detection (PSD) scheme is a new approach to data processing algorithms for electrical substitution radiometers, with impact on the uncertainty analysis, compared to the time-based demodulation approach used by all other TSI instruments [2]. Thus it represents a potential source of the 0.35 % TSI scale difference in the minds of several participants at the TSI workshop. It can be viewed as a new way to demodulate the (nearly) square-wave substitution power vs. time data that results from an active cavity radiometer viewing a stable source while cycling its shutter at a constant frequency and 50 % duty cycle. Traditional instruments analyze this in the time domain, for instance by subtracting the settled shutter-open average power value from the settled shutter-closed average power value for each shutter cycle as a function of time. TIM’s PSD algorithm analyzes this instead in the frequency domain by transforming the power values into the frequency domain and referencing to the shutter frequency. It offers an advantage of rejecting interfering signals at higher (noise) and lower (drift) frequencies than the shutter-cycle frequency. It requires corrections for the shutter waveform and the power-control servo-loop gain, both of which are done based upon measurements on the TIM instrument.

In his presentation on Day 1, Greg Kopp showed a comparison of on-orbit SORCE/TIM data analyzed using the PSD and a DC method similar to that used by other TSI instrument teams. The difference was less than 0.025 %, much smaller than the observed 0.35 % discrepancy between the SORCE/TIM and the other TSI instruments. For this comparison, there was no attempt to evaluate the best estimates for the non-equivalence and gain for the DC method, so the actual difference could be less than 0.025 %.

### 3.7 Joule Heating

In addition to scattering, another potential source of error not discussed in full detail at the workshop by all instrument teams was Joule heating in the current leads for the electrical substitution heater. Some of the heat dissipated in these leads goes through the receiver cavity before going to the heat sink, whereas the rest goes directly to the heat sink. It is necessary, in all existing TSI instruments, to correct for the fraction of modulated heater power that is measured but leaks away into the heat sink (or, alternatively, that is not measured but is deposited on the receiver). Note that one reason that cryogenic electrical substitution radiometers revolutionized the optical power metrology field thirty years ago is that superconducting leads can be used, where there is no Joule heating so this effect is non-existent. In Table 1, this uncertainty component is included in the “Standard Ohm + Leads’’ term if it was available. As with several of the other potential sources of error, the proposed TSI laboratory intercomparison discussed below will probe this effect, albeit in combination with the others, to experimentally determine if it has been properly corrected in the TSI instruments.

## 4. Recommended Path Forward

Possible paths forward discussed at the workshop include performing comprehensive scattering calculations analogous to what was done for diffraction, and measurement intercomparisons with the laboratory versions of existing flight units. Two intercomparison proposals were discussed. One proposal is to perform a laboratory intercomparison using lasers at NIST. The other is to perform a mountain-top intercomparison using the Sun. These two proposals can be viewed as complementary, so that both could eventually be performed. However, the laboratory intercomparison is probably less costly, has the lowest uncertainty, provides a direct link to the SI, and is the easiest and quickest to implement, so it is the recommended place to start. In the following we first elaborate a bit more on the mountain-top proposal, then give a much more lengthy description of the NIST TSI laboratory inter-comparison plan. This discussion begins with an introduction, continues with a description of the proposed optical configuration and experimental procedure, and concludes with estimates of the uncertainty budget.

### 4.1 Mountain-Top Intercomparison

The mountain-top proposal is to use existing facilities at Table Mountain in California to have multiple TSI instruments (copies of existing flight units) view the Sun at the same time. The instruments would be in a common vacuum chamber, looking at the Sun through the chamber window and the Earth’s atmosphere. Correction for effects such as the halo around the Sun that results from atmospheric scattering would limit the standard uncertainty to something like 0.2 % (*k* = 1) at the very lowest, which presents a risk of this approach for discerning the 0.35 % scale difference. Scattering of a broadband light source (the Sun) in the plane-parallel window of the vacuum chamber will probably increase the uncertainty above this. Also, the issue of how to connect such proposed intercomparison measurements to a national radiometric scale was not substantially dealt with at the workshop; one approach discussed was the development of a cryogenic TSI instrument that could work at the Table Mountain facility and also be calibrated against the NIST scale. This is useful because a relative intercomparison between TSI instruments would not resolve the question raised in Fig. 1: it is essential to have high accuracy connection to a national radiometric scale in order for such intercomparisons to be useful.

### 4.2 TSI Laboratory Intercomparison

The proposed NIST TSI laboratory intercomparison plan is for each TSI instrument team to bring a representative TSI radiometer, one at a time, to the NIST calibration facilities in Gaithersburg, MD, for laboratory intercomparison against the NIST irradiance scale. Representatives from each of four TSI instrument teams, TIM, ACRIM, DIARAD, and PMO6, would provide an instrument that has a design basically equivalent to their respective flight instrument(s). The consensus from the TSI uncertainty workshop and discussions afterwards was that each team already has such an instrument, and is ready and willing to participate. ERBE is not included in this list since it has no such instrument. Also, each of these TSI laboratory instruments will have its own absolute scale which will have been determined by each team using procedures basically equivalent to those that were used to calibrate the flight instruments for TSI. During the intercomparison at NIST, each instrument will measure a NIST-developed irradiance source at TSI power levels, and NIST will measure the irradiance of the same source. The results will be then be compared.

The primary objective is to perform the irradiance intercomparison at one wavelength (532 nm) with sufficiently high accuracy so as to determine if there are any differences between the individual TSI instrument scales and the NIST scale. Thus each laboratory TSI instrument will be operated in a manner consistent with achieving uncertainty levels that are similar to the corresponding flight instrument. A major assumption in the applicability of the results to helping to understand the current 0.35 % spread in TSI is that the scales placed on the participating instruments by the individual teams are truly representative of the flight instruments. This applies to both the mean and the uncertainty of the measurements made by each TSI instrument. NIST will provide a measurement of the laboratory irradiance source against the NIST irradiance responsivity scale with an uncertainty goal of 0.05 % (*k* = 1). Also, the irradiance source will be controlled, spatially and temporally, such that the source components of uncertainty that add to any of the TSI laboratory instrument measurements will be below 0.05 % (*k* = 1).

There are three related secondary objectives. One is to perform power-mode measurements to compare the native power-mode scales with the NIST power mode scale. The second is to measure the response as a function of beam diameter, which may provide insight into certain scattering and diffraction effects that may help understand the TSI scale differences. The third is to test the assumption that one wavelength is adequate for the irradiance intercomparison by measuring the reflectance of representative black cavities from each instrument team over the full spectral range.

To enable the measurement of response versus beam diameter, the proposed experimental arrangement provides the ability to vary the diameter of the optical beam used to simulate the Sun. “Power mode’’ is when the beam diameter is less than the defining aperture of the TSI instrument (or NIST irradiance standard); “irradiance mode’’ is when the input beam diameter is greater than the diameter of the defining aperture. This mode continues as the beam diameter becomes greater than the diameter of the field-of-view-limiting aperture. The irradiance intercomparison will closely examine the results from the largest beam diameter, which most resembles solar illumination, and has a diameter greater than the diameter of the largest field-of-view-limiting aperture of the TSI instruments. By performing measurements of instrument response as the diameter of the beam is increased from power mode, to filling the defining aperture, to filling the field-of-view-limiting aperture, the power mode and response-vs-beam-diameter study objectives can be met.

The measurements will be conducted at the laser wavelength of 532 nm. This wavelength was chosen since it is near the peak of the solar spectrum, a sufficiently high-power (10 W) laser already exists at the NIST facility at this wavelength, and use of a single laser line will enable laboratory optics with high performance antireflection coatings to be used in the construction of the laboratory irradiance source, significantly reducing uncertainties associated from scattered light and ghost images from the source. Also, beam-splitter characterization and window transmittance measurements, at the uncertainty levels required, are much easier at one or a few laser wavelengths than with broadband sources. This means that the irradiance intercomparison itself will not probe any hypothetical uncorrected spectral dependence of the TSI instruments. Thus we are making the assumption that any relative spectral errors in the native scales are not large enough to cause the 0.35 % TSI spread. This assumption is reasonable because all TSI instruments use black cavities that have in some cases been measured for reflectance at wavelengths spanning the spectrum of interest for TSI. The instrument scales already incorporate, where significant, the known small deviations from perfect absorbance. Thus spectral issues related to cavity absorptance are probably very small to begin with (at least for pre-flight cavities) and have already been corrected. Therefore it does not seem justified, at least for this first-ever comprehensive TSI laboratory scale intercomparison, to incur the huge (> 10 ×) additional cost that would be required to achieve useful broadband irradiance measurements. However, to test the assumption that spectral issues are not significant, the spectral reflectance of representative black cavities from each instrument team will be measured over the spectral range from 250 nm to 5000 nm using tunable lasers, integrating spheres, and proven, existing techniques at the NIST Spectral Irradiance and Radiance Responsivity Calibrations with Uniform Sources (SIRCUS) facility.

#### 4.2.1 Optical Configuration

**Fig. 4:**
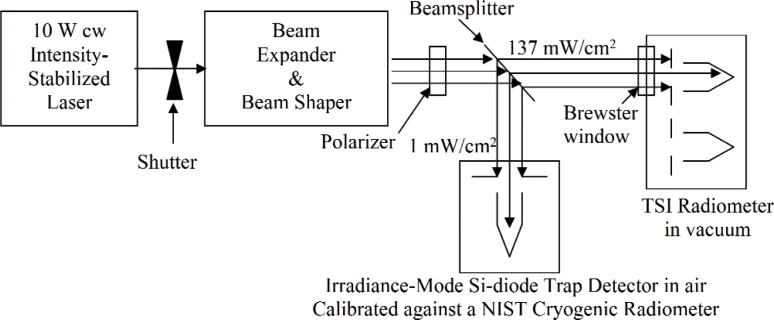
Proposed TSI laboratory intercomparison basic optical configuration.

The basic optical layout for the laboratory intercomparison is depicted schematically in Fig. 4. A 10 W cw laser beam is intensity stabilized and sent through a two-lens telescope that acts a beam expander, providing an output beam diameter of up to 20 mm. This beam is sent through a beamsplitter which transmits most of it, adjustable to be at the TSI irradiance level, to the TSI radiometer. The reflected beam is sent to the NIST standard for irradiance measurement. The beamsplitter ratio, reflectance/transmittance, measured in a separate step, is applied to the NIST standard detector measurements to determine the irradiance sent to the TSI radiometer. During the same time interval, the TSI radiometer measures the irradiance on its native scale.

There are a few variations in the details of the basic scheme that are currently being explored by NIST. Depending on the type of attenuator employed in the intensity stabilizer, it may be necessary to use a spatial filter before the beam expander. Also, the laser provides a beam having a Gaussian cross-sectional irradiance profile. As an option, this can be passed through a refractive beam shaper that converts the Gaussian profile to a flat-topped profile that more closely approximates the Sun. The resulting uniformity of the output beam, along with its effect on the uncertainty of the measurements proposed, is currently being determined by NIST using a prototype experimental setup and a commercially available refractive beam shaper [6].

Also, a polarizer will be placed in the beam prior to the beamsplitter. This is to ensure vertical polarization that is needed for near-unity transmittance through the Brewster-angled window for the vacuum chamber that houses the TSI instrument. Use of Brewster-angled windows is the normal practice with cryogenic radiometers, and the NIST experience with measuring the transmittance of a Brewster-angled window to a vertically-polarized beam is that it can be determined with uncertainty substantially less than 0.01 %. This is mainly due to the lack of substantial scattering in the Brewster-angle geometry.

For enabling the test of instrument response as a function of beam diameter, there are two alternatives planned for achieving the variable beam diameter. The simplest way is to use a variable iris just prior to the beamsplitter as a beam-limiting aperture. This may lead to undesirable diffraction and scattering from the iris edge. An alternative is to design the beam expander for variable magnification, from a few mm for the power-mode to a maximum diameter of about 20 mm for irradiance-mode. The beam diameter can be varied in steps by successively replacing one of the lenses of the beam expanding telescope with lenses of successively longer focal length placed proportionally farther away.

#### 4.2.2 Experimental Procedure

The irradiance will be measured by NIST using a Si-diode trap detector fitted with a precision aperture. The power responsivity of the trap is measured up to 1 mW power level against a cryogenic radiometer by NIST at the 0.02 % uncertainty level. The aperture area is measured using the NIST aperture area measurement facility at the 0.01 % level or below. The nominal value of the aperture area will be 0.5 cm^2^ during the TIM and ACRIM measurements, so as to match the defining aperture area of those instruments. The spatial uniformity of the trap over the aperture area is mapped, and is at the 0.01 % level. There is negligible back-reflectance from the trap that would scatter from the back of the aperture. Thus, the power responsivity and aperture area are combined to give an irradiance responsivity for the trap when used in irradiance mode (aperture overfilled), as is the standard procedure at the NIST SIRCUS facility.

A shutter, shown in Fig. 4, will be closed to determine the background level for the trap measurement. This shutter will provide a way of determining the dark level for TSI instrument measurements by recording the signal when it is closed. Each TSI instrument will be mounted in a vacuum chamber. The collimated laser beam from the NIST source will enter the vacuum chamber through a window mounted at Brewster’s angle, as determined by adjusting the window angle until the reflection from its front surface is null or minimized. The window transmittance will be near unity for the p-polarized laser beam used, and the actual transmittance will be measured by NIST. A method will be developed by NIST for easily and routinely performing the window transmittance measurement. For beam diameters of 2 mm to 3 mm, this is done routinely at the NIST Primary Optical Watt Radiometer (POWR) facility with uncertainty less than 0.01 %.

The TSI instrument will be aligned both translation-ally and angularly. It will be mounted on a motorized vertical-horizontal translation stage that will enable translational alignment by maximization of the signal while the instrument is under vacuum. Angular alignment will be achieved during mounting of the TSI instrument in the chamber, by minimizing the deviation of a low-power laser beam retro-reflected from a reference plane from the instrument, the reference plane being one that is parallel to the defining aperture plane.

Response measurements will be made with the TSI instrument and the trap simultaneously. Measurements will be made over a range of beam diameters, from 3 mm to 15 mm. For beam diameters near 3 mm, the beam will underfill the apertures and so the measurements will be used to intercompare the native power scales of the TSI instruments with the NIST power-responsivity scale. For beam diameters near 15 mm, the beam will overfill the defining aperture of the TSI instruments and the trap, and so the measurements will be used to intercompare the native irradiance scales of the TSI instruments with the NIST irradiance responsivity scale. For beam diameters large enough to overfill the defining aperture of the TSI instrument but still small enough to underfill the field-of-view-limiting aperture of the TSI instrument, any variation of response not accounted for by imperfect beam spatial uniformity may indicate effects of scattering or diffraction.

NIST will automate the data collection and analysis processes to the extent that is practical, using software routinely used at the POWR facility and modifying it to interface with each TSI instrument.

#### 4.2.3 Uncertainty Budget

Predictions for the values of the major sources of uncertainty that will affect the irradiance comparison are listed in Table 4, and each source is discussed below. We note the values may be specific to the experimental parameters and underlying assumptions considered. The *k* = 1 combined standard uncertainty is estimated as the root of the sum of the squares (RSS) value of components, and is estimated as < 500 × 10^−6^. This corresponds to a 95 % confidence level (*k* = 2) uncertainty value of 0.1 %, which should be adequate to resolve the 0.35 % difference between the TSI scales.

##### 4.2.3.1 Trap Absolute Irradiance Responsivity

This includes the typical absolute responsivity of 200 × 10^−6^ (0.02 %) uncertainty of trap calibrations against NIST cryogenic radiometers at < 1 mW power levels, and allows for additional uncertainty from irradiance trap aperture area and trap spatial uniformity.

##### 4.2.3.2 Trap Signal Variations

This accounts for the random noise and drift that will be seen during the trap phase of the measurements of the irradiance beam. It will be determined by the standard deviation of the mean of repeated measurements. It can be in principle reduced by increasing the number of measurements averaged, but practical time constraints limit it to the value shown based upon experience. It includes trap background subtraction, which is routinely done by closing the shutter and contributes negligibly to the uncertainty since the silicon detectors are not sensitive to background thermal-infrared drift.

##### 4.2.3.3 TSI Instrument Signal Variations

This accounts for the random noise and drift that will be seen during the TSI instrument phase of the measurements of the irradiance beam. It will be determined by the standard deviation of the mean of repeated measurements. It can in principle be reduced by increasing the number of measurements averaged, but practical time constraints limit it to the value shown based upon experience. Note that there will be some variation of the values from different TSI instruments for this effect.

##### 4.2.3.4 TSI Instrument Background Subtraction

The shutter will be used to take dark measurements with the TSI instrument, which gives a correction related to the thermal-infrared background seen by the TSI instruments. However, it is recognized that this process may not exactly replicate the background subtraction performed on orbit by some TSI instruments, such as slewing to view deep space. For example, there could be effects related to shutter heating that may not be simulated properly. This component of uncertainty accounts for additional systematic uncertainty associated with such effects. Its value is an estimate, and it will probably vary between the instruments. There are probably ways to refine this uncertainty value during the testing based on characterizations using both the source shutter and the TSI instrument(s) internal shutter(s).

##### 4.2.3.5 Irradiance Temporal Stability

The laser power will be stabilized by an active high-power laser intensity stabilizer. The estimate is based on experience at POWR and other facilities at NIST, where laser intensity stabilization to better than 50 × 10^−6^ is routine.

##### 4.2.3.6 Window Transmittance

The estimate is based upon POWR experience for a p-polarized collimated laser beam of 3 mm diameter through a clean Brewster-angled window. Work at NIST will determine the degree to which this still holds for beam diameters up to 15 mm.

##### 4.2.3.7 Beamsplitter Ratio

This will involve turning the laser power down so that < 1 mW is transmitted through the beamsplitter, then positioning the trap alternately in the transmitted and reflected positions to measure the ratio. Though there are some techniques common with the window transmittance measurement, it is recognized that this is a more difficult characterization so additional uncertainty is allowed for in the budget. Also, additional uncertainty needs to be included to allow for small effects which might make the ratio change between the 1 mW power level at which it is measured versus the 75 mW power level at which it is used. For example, the attenuator used may change the spatial uniformity or polarization slightly. Also, the linearity of the trap detector, while in principle very good over the dynamic range required, must be checked, and any uncertainties in the linearity would contribute here.

##### 4.2.3.8 Beam Sampling Equivalence

The laser beam profile will not match the Sun perfectly. Work is currently in progress at NIST to determine if it is better to use the beam shaper, which in principle supplies a top-hat profiled beam that best simulates solar irradiance, or a Gaussian profiled beam which is the more natural output of a laser. The major source of uncertainty from non-uniformity is ensuring that both the NIST trap and the TSI radiometer sample the same part of the beam. For purposes of providing the number in Table 4, a Gaussian beam was assumed that has a beam waist diameter of 50 mm, and a nominal TSI instrument defining aperture diameter of 8 mm (corresponding to 0.5 cm^2^) was used. Then if the aperture areas of the trap and the TSI instrument differ by 1 % of each other, and they are each centered on the same Gaussian profile, the difference between flat profile and Gaussian profile is the 64 × 10^−6^ quoted in Table 4. By mapping the profile to confirm its profile, correction for this effect can be implemented and the associated uncertainty reduced.

Since the TSI instrument will be translationally aligned to maximum signal, there need be no uncertainty for off-centered apertures as long as the noise of the TSI instrument is small enough and the step size of the stage is fine enough. However, there could be a small component of additional uncertainty for spatial non-uniformity of the power responsivity within the aperture of the TSI instrument as it is convolved with the spatial non-uniformity of the beam.

##### 4.2.3.9 Angular Alignment

This was estimated by assuming that the TSI instrument aperture can be aligned normal to the beam within 10 mrad, which should be possible using the retro-reflection technique. The error source is the usual cos θ term for the area of the aperture projected normal to the beam.

## 5. Related Work

As discussed by Claus Fröhlich at the TSI workshop, a power-mode scale intercomparison was performed by NPL/PMOD, and found agreement between the World Radiometric Reference (WRR) as applied to a PMO TSI instrument, and the SI radiometric scale as established by the United Kingdom’s National Physical Laboratory (NPL), to within 109 × 10^−6^, with an uncertainty of 1600 × 10^−6^ at the 95 % confidence level [7,8,9]. It differed from the proposed NIST laboratory intercomparison in the following ways. It did not include TIM, ACRIM, or DIARAD. The TSI instrument (PMO6) was operated in air, rather than in vacuum. It compared power-mode only, with a 4 mm diameter beam that underfilled the 5 mm diameter defining aperture of the PMO6 instrument. It was similar to the proposed intercomparison in that it used laser lines, silicon trap detectors, and it used the beamsplitter approach to reduce the power level to be compatible with the silicon trap detector. Also, the silicon trap detector was calibrated on the NPL power responsivity scale, as established on one of the NPL cryogenic electrical substitution radiometers.

**Fig. 5:**
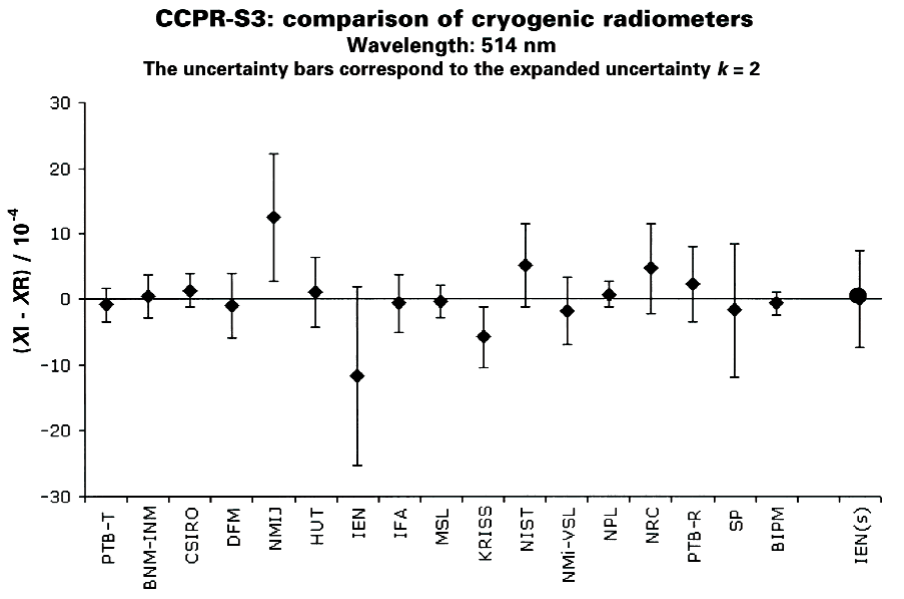
Results of the CCPR supplementary comparison of cryogenic radiometers, CCPR-S3, at the wavelength 514 nm, taken from the BIPM comparison database [10]. The quantity compared was the radiant power responsivity scale as applied to silicon photodiode trap detectors that were sent between the laboratories. The participants were all national measurement institutes, including NIST and NPL, as plotted on the horizontal axis. The results are presented as the relative difference of the participant (XI) to a weighted mean (XR). The value for IEN(S) refers to a supplementary bilateral comparison performed after the original comparison but following the same protocol [11].

The cousins of TSI active cavity radiometers are the cryogenic electrical substitution radiometers in use by the world’s electro-optical metrology community for establishing the optical watt. These differ in just a few details from the TSI instruments: they generally work in a laboratory rather than in space, they generally operate at temperature near 5 K to 20 K rather than above 300 K, they generally measure less than 1 mW rather than 20 mW to 70 mW, and they generally measure laser power that underfills the aperture rather than solar power that overfills the aperture. Cryogenic radiometers were intercompared internationally in the late 1990s by sending trap detectors around to more than a dozen national metrology institutes and comparing their results for power mode responsivity calibration of the traps [10,11]. Agreement was generally within 0.02 %, as shown in Fig. 5. NIST has since updated to new cryogenic electrical substitution radiometers and compared them internally, finding agreement within the 0.02 % (*k* = 1) uncertainty level [12]. The key point to appreciate here is that it is indeed possible, at least for cryogenic electrical substitution radiometers, to achieve radiometric accuracy much better than the 0.35 % TSI scale difference.

## Figures and Tables

**Table 1:** Uncertainty budgets (*k* = 1) for the absolute scales of TSI instruments, with standard uncertainty components and the combined standard uncertainty, called “Total RSS” (root of the sum of the squares), given in parts per million (× 10^−6^). A value of 1 indicates the component is multiplicative in the TSI determination. The table represents best-effort estimates as reported by the workshop participants. As such, it is somewhat incomplete and the estimates are not necessarily correct. “N/R” means “not reported” and “N/A” means “not applicable.”

Instrument Component	ACRIM III		ERBE		DIARAD		PMO6V		TIM	
	Value	Unc.	Value	Unc.	Value	Unc.	Value	Unc.	Value	Unc.
Aperture Area	1000000	280	1000000	833	1000000	400	1000000	501	1000000	30
Diffraction	0	1295	0	200	776	N/R	1000	100	452	47
Cavity Reflectance	500	200	120	7	250	300	330	70	170	54
Non-Equivalence	N/R	N/R	N/R	N/R	0	200	2900	500	7	23
Standard Volt + DAC	1000000	101	1000000	498	1000000	150	1000000	100	1000000	186
Standard Ohm + Leads	1000000	200	1000000	23	1000000	N/R	1000000	30	1000000	17
Thermal Background (Dark)	N/R	N/R	4400	33	N/R	N/R	N/R	N/R	2693	10
Scattered Light	N/R	N/R	N/R	N/R	N/R	N/R	320	100	100	25
Servo Gain	N/R	N/R	N/R	N/R	329	N/R	N/R	N/R	16129	0
Shutter Waveform	N/A	0	N/A	0	N/A	0	N/A	0	100	1
Correction to 1 AU	33537	0.1	33537	33	N/R	N/R	N/R	N/R	33537	0.1
Doppler Effect	0	57	0	57	N/R	N/R	N/R	N/R	57	0.7
Pointing	N/R	N/R	N/R	133	N/R	N/R	N/R	N/R	N/R	10
Measurement Repeatability	N/A	N/R	N/A	66.7	N/A	N/R	N/A	223.6	N/A	1.5
On-orbit Degradation	300	12	~0	N/R	366	73	2930	87	90	10

Total RSS		1359.8		1005.0		563.8		771.0		205.8

**Table 2:** Stated uncertainties (*k* = 1) compared with observed cavity variations. The stated value for the uncertainty for ERB on NIMBUS 7 was the design requirement. For ACRIM II and ACRIM III, the stated uncertainty was estimated from the cavity variation.

Instrument	TSI Value (W/m^2^)	Stated Uncertainty (× 10^−6^)	Cavity Variation (× 10^−6^)
ERB (NIMBUS 7)	1371.9	5000	N/A
ACRIM I	1367.5	1000	510
ACRIM II	1364.2	2000	2045
ACRIM III	1366.1	1000	1034
ERBE	1365.2	833	N/A
VIRGO-PMO6V	1365.7	1204	316
VIRGO-DIARAD	1366.4	470	2100
TIM	1361.0	350	266

**Table 3:** Results of NIST diffraction calculations. The reported correction is multiplicative with the area of the defining aperture. The Sun was modeled spectrally as a 5900 K blackbody. *R_a_* is the radius of the front aperture (closest to the Sun), *R_d_* is the radius of the rear aperture, and *d_d_* is the distance between the two apertures.

Instrument		*R_a_* (mm)	*d_d_* (mm)	*R_d_* (mm)	Correction	Correction Applied?
PMO6V		4.25	95.4	2.5	1.001280	Yes
DIARAD		6.52	144	4.0015	1.000833	Yes
ERBE ACRIM		12.09	100.8	4.039	1.000209	No
	Baf1	6.6548	150.4696	3.9878	1.000828	
	Baf2	6.3119	76.3524	3.9878	1.000466	
	Total				1.001295	No
TIM		3.9894	101.6	7.62	0.999582	Yes

**Table 4:** Uncertainty budget for laboratory irradiance comparison.

Source of Uncertainty Component	Predicted Value of Uncertainty (× 10^−6^, *k* = 1)
Trap absolute irradiance responsivity	275
Trap signal variations	100
TSI instrument signal variations	100
TSI instrument background subtraction	200
Irradiance temporal stability	50
Window transmittance	100
Beamsplitter ratio	300
Beam sampling equivalence	64
Angular alignment	50

RSS Total	495
